# Weighted norm inequalities for multilinear Calderón-Zygmund operators in generalized Morrey spaces

**DOI:** 10.1186/s13660-017-1325-z

**Published:** 2017-02-22

**Authors:** Panwang Wang, Zongguang Liu

**Affiliations:** Department of Mathematics, China University of Mining and Technology, Beijing, 100083 P.R. China

**Keywords:** 42B20, multilinear Calderón-Zygmund operators, commutators, weighted Morrey spaces

## Abstract

In this paper, the authors study the boundedness of multilinear Calderón-Zygmund singular integral operators and their commutators in generalized Morrey spaces.

## Introduction

Let *T* be a multilinear operator initially defined on the *m*-fold product of Schwartz spaces and taking values into the space of tempered distributions, i.e.,

$$ T : \mathcal{S}\bigl(\mathbb{R}^{n}\bigr)\times\cdots\times\mathcal {S}\bigl(\mathbb{R}^{n}\bigr)\rightarrow\mathcal{S}'\bigl( \mathbb{R}^{n}\bigr). $$

In [1], it is said that a function *K* belongs to the class *m*-$\operatorname{CZK}(A,\varepsilon)$ if

1. $|K(y_{0},y_{1},\ldots,y_{m})|\leqslant\frac{A}{(\sum_{k,l=0}^{m}|y_{k}-y_{l}|)^{mn}}$,
2. if $|y_{j}-y_{j}'|\leqslant\frac{1}{2}\max_{0\leqslant k\leqslant m}|y_{j}-y_{k}|$,
  $$\bigl\vert K(y_{0},\ldots,y_{j},\ldots,y_{m})-K \bigl(y_{0},\ldots,y_{j}',\ldots,y_{m} \bigr)\bigr\vert \leqslant \frac{A|y_{j}-y_{j}'|^{\varepsilon}}{(\sum_{k,l=0}^{m}|y_{k}-y_{l}|)^{mn+\varepsilon}} $$
  for some $\varepsilon>0$ and $j=0,1,2\ldots, m$.

The operator *T* is said to be an *m*-linear Calderón-Zygmund operator if there exists a function $K\in m\mbox{-}\operatorname{CZK}(A,\varepsilon)$ defined away from the diagonal $y_{0}=y_{1}=y_{2}\cdots=y_{m}$ in $(\mathbb{R}^{n})^{m+1}$ such that

1.1 $$ T(f_{1},\ldots,f_{m}) (x)= \int_{(\mathbb{R}^{n})^{m}}K(x,y_{1},\ldots ,y_{m})f_{1}(y_{1}) \cdots f_{m}(y_{m}) \, \mathrm{d}y_{1}\cdots\, \mathrm{d}y_{m} $$

for $x\notin\bigcap_{j=1}^{m} \operatorname{supp} f_{j}$, and that *T* extends to a bounded multilinear operator from $L^{q_{1}}\times\cdots\times L^{q_{m}}$ to $L^{q}$ for some $1\leqslant q_{j}<\infty$ with $\frac{1}{q}=\frac{1}{q_{1}}+\cdots +\frac{1}{q_{m}}$.

It was shown in [1] that if $\frac{1}{r_{1}}+\cdots+\frac {1}{r_{m}}=\frac{1}{r}$, then an *m*-linear Calderón-Zygmund operator satisfies

$$ T:L^{r_{1}}\times\cdots\times L^{r_{m}}\rightarrow L^{r} $$

when $1< r_{j}<\infty$ for $j=1,\ldots,m$ and

$$ T:L^{r_{1}}\times\cdots\times L^{r_{m}}\rightarrow L^{r,\infty} $$

when $1\leq r_{j}<\infty$ for $j=1,\ldots,m$ and at least one $r_{j}=1$. In particular,

$$ T:L^{1}\times\cdots\times L^{1}\rightarrow L^{1,\infty}. $$

The theory of multiple weight associated with *m*-linear Calderón-Zygmund operators was developed by Lerner et al. [2]. Let $1< p_{j}<\infty$ for $j=1,\ldots,m$, $\frac{1}{p}=\frac {1}{p_{1}}+\cdots+\frac{1}{p_{m}}$ and $\vec{p}=(p_{1},\ldots,p_{m})$, we say $\vec{\omega}\in A_{\vec{p}}$ if

$$ \sup_{B} \biggl(\frac{1}{|B|} \int_{B}v_{\vec{\omega}} \biggr)^{1/p}\prod _{j=1}^{m} \biggl(\frac{1}{|B|} \int_{B} \omega_{j}^{1-p{'}_{j}} \biggr)^{1/p{'}_{j}}< \infty, $$

where *B* is the ball in $\mathbb{R}^{n}$ and $v_{\vec{\omega}}=\prod_{j=1}^{m}\omega_{j}^{p/p_{j}}$. They showed that if $\vec{\omega}\in A_{\vec{p}}$ then

1.2 $$ \bigl\Vert T(\vec{f})\bigr\Vert _{L^{p}(v_{\vec{\omega}})}\leqslant C\prod _{j=1}^{m}\Vert f_{j}\Vert _{L^{p_{j}}(\omega_{j})} . $$

If $1\leqslant p_{j}<\infty$ for $j=1,\ldots,m$ and at least one of the $p_{j}=1$, they also proved

1.3 $$ \bigl\Vert T(\vec{f} )\bigr\Vert _{L^{p,\infty}(v_{\vec{\omega}})}\leqslant C \prod_{j=1}^{m}\Vert f_{j} \Vert _{L^{p_{j}}(\omega_{j})} . $$

Let $\vec{b}=(b_{1},\ldots,b_{m})$ be a vector-valued locally integrable function. If $\vec{b}=(b_{1},\ldots,b_{m})$ in $(\mathit{BMO})^{m}$, we denote $\|\vec{b}\|_{(\mathit{BMO})^{m}}=\sup_{j=1,\ldots,m}\|b_{j}\|_{\mathit{BMO}}$ (see [2]). The commutator generated by an *m*-linear Calderón-Zygmund operator *T* and a $(\mathit{BMO})^{m}$ function *b⃗* is defined by

1.4 $$ T_{\vec{b}}(f_{1},\ldots,f_{m})=\sum _{j=1}^{m}T_{\vec{b}}^{j}(\vec{f} ), $$

where each term is the commutator of $b_{j}$ and *T* in the *j*th entry of *T*, that is,

$$ T_{\vec{b}}^{j}(\vec{f} )=b_{j}T(f_{1}, \ldots,f_{j},\ldots ,f_{m})-T(f_{1}, \ldots,b_{j}f_{j},\ldots,f_{m}) . $$

Pérez and Torres [3] proved that if $\vec{b}\in(\mathit{BMO})^{m}$ then

$$ T_{\vec{b}} : L^{p_{1}}\times\cdots\times L^{p_{m}} \rightarrow L^{p} $$

for $1< p_{j}<\infty$ and $1< p<\infty$ with $\frac{1}{p}=\frac {1}{p_{1}}+\cdots+\frac{1}{p_{m}}$, where $j=1,\ldots,m$. In [2], the authors proved that if $\vec{\omega}\in A_{\vec{p}}$ and $\vec{b}\in(\mathit{BMO})^{m}$, then

1.5 $$ \bigl\Vert T_{\vec{b}}(\vec{f})\bigr\Vert _{L^{p}(v_{\vec{\omega}})} \leqslant C\Vert \vec {b}\Vert _{(\mathit{BMO})^{m}}\prod _{j=1}^{m}\Vert f_{j}\Vert _{L^{p_{j}}(\omega_{j})} $$

for $1< p_{j}<\infty$ with $\frac{1}{p}=\frac{1}{p_{1}}+\cdots+\frac {1}{p_{m}}$, where $j=1,\ldots,m$.

Feuto [4] introduced the generalized weighted Morrey space $(L^{p}(\omega),L^{q} )^{\alpha}$. Let $1\leqslant p\leqslant \alpha\leqslant q\leqslant\infty$ and *ω* be a weight. The space $(L^{p}(\omega),L^{q} )^{\alpha}$ was defined to be the set of all measurable functions *f* satisfying $\|f\|_{ (L^{p}(\omega ),L^{q} )^{\alpha}}<\infty$, where

$$ \|f\|_{{ (L^{p}(\omega),L^{q} )^{\alpha}}}=\sup_{r>0}{}_{r}{\|f \|}_{{ (L^{p}(\omega),L^{q} )^{\alpha}}} $$

with

$$ {}_{r}{\Vert f\Vert }_{{ (L^{p}(\omega),L^{q} )^{\alpha}}}:= \biggl[ \int_{\mathbb{R}^{n}} \bigl({\omega\bigl(B(y,r)\bigr)^{1/\alpha-1/p-1/q}} \Vert f\chi_{B(y,r)}\Vert _{L^{p}(\omega )} \bigr)^{q} \, \mathrm{d}y \biggr]^{1/q}. $$

When $\omega\equiv1$, the space $(L^{p},L^{q} )^{\alpha }$ was introduced in [5]. If $p<\alpha$ and $q=\infty$, the space $(L^{p}(\omega ),L^{\infty} )^{\alpha}$ is just the weighted Morrey space $L^{p,\kappa}(\omega)$ with $\kappa=1-p/\alpha$ defined by Komori and Shirai [6].

Similarly, the weak space $(L^{p,\infty}(\omega),L^{q} )^{\alpha}$ is defined with

$$ {}_{r}{\Vert f\Vert }_{{ (L^{p,\infty}(\omega),L^{q} )^{\alpha }}}:= \biggl[ \int_{\mathbb{R}^{n}} \bigl(\omega\bigl(B(y,r)\bigr)^{1/\alpha-1/p-1/q} \Vert f\chi_{B(y,r)}\Vert _{L^{p,\infty}(\omega )} \bigr)^{q}\, \mathrm{d}y \biggr]^{1/p}. $$

When $p=1$, the space $(L^{1,\infty}(\omega),L^{q} )^{\alpha}$ was introduced in [4].

Feuto proved in [4] that Calderón-Zygmund singular integral operators, Marcinkiewicz operators, the maximal operators associated to Bochner-Riesz operators and their commutators are bounded on $(L^{p}(\omega),L^{q} )^{\alpha}$.

In this paper, we aim to study the boundedness of multilinear singular integral operators on the product of generalized Morrey spaces. Inspired by the above mentioned works, we state our main results as follows.

### Theorem 1.1

*Let* *T* *be an m*-*linear Calderón*-*Zygmund operator*, $\frac{1}{p}=\frac {1}{p_{1}}+\cdots+\frac{1}{p_{m}}$ *and* $\vec{\omega}\in A_{\vec{p}}$.

1. *If* $1< p_{j}<\infty$, $j=1,\ldots,m$ *and* $p\leqslant \alpha< q\leqslant\infty$, *then*
  $$\bigl\Vert T\vec{(f)}\bigr\Vert _{(L^{p}(v_{\vec{\omega}}),L^{q})^{\alpha}}\lesssim \prod _{i=1}^{m}\Vert f_{i}\Vert _{({L^{p_{i}}(\omega_{i}),L^{qp_{i}/p}})^{\alpha p_{i}/p}}. $$
2. *If* $1\leqslant p_{j}<\infty$, $j=1,\ldots,m$ *and at least one of* $p_{j}=1$, $p\leqslant\alpha< q\leqslant\infty$, *then*
  $$\bigl\Vert T\vec{(f)}\bigr\Vert _{(L^{p,\infty}(v_{\vec{\omega}}),L^{q})^{\alpha }}\lesssim\prod _{i=1}^{m}\Vert f_{i}\Vert _{({L^{p_{i}}(\omega _{i}),L^{qp_{i}/p}})^{\alpha p_{i}/p}}. $$

### Theorem 1.2

*Let* $T_{\vec{b}}$ *be a multilinear commutator*, $\vec{b}\in (\mathit{BMO})^{m}$, $\frac{1}{p}=\frac{1}{p_{1}}+\cdots+\frac{1}{p_{m}}$ *with* $1< p_{j}<\infty$ *and* $\vec{\omega}\in A_{\vec{p}}$. *If* $p\leqslant \alpha< q\leqslant\infty$, *then*

$$\bigl\Vert T_{\vec{b}}(\vec{f})\bigr\Vert _{(L^{p}(v_{\vec{\omega}}),L^{q})^{\alpha }}\lesssim \Vert \vec{b}\Vert _{(\mathit{BMO})^{m}}\prod_{i=1}^{m} \Vert f_{i}\Vert _{({L^{p_{i}}(\omega_{i}),L^{qp_{i}/p}})^{\alpha p_{i}/p}}. $$

### Remark 1.3

When $m=1$, Theorem 1.1 is just Theorem 2.1 in [4] and Theorem 1.2 is just Theorem 2.5 in [4].

## Notations and preliminaries

We first recall the definition of $A_{p}$ weight. A nonnegative locally integrable function *ω* belongs to $A_{p}$ ($p>1$) if

$$ \sup_{B} \biggl(\frac{1}{|B|} \int_{B}\omega(x) \, \mathrm{d}x \biggr) \biggl( \frac{1}{|B|} \int_{B}\omega(x)^{1-p'}\, \mathrm{d}x \biggr)^{p-1}< \infty, $$

where $p'$ is the conjugate index of *p*, i.e., $1/p+1/p'=1$. We say that $\omega\in A_{1}$ if there is a constant $C>0$ such that

$$ \frac{1}{|B|} \int_{B}\omega(x)\, \mathrm{d}x\leqslant C\inf _{x\in B}\omega(x) $$

for any ball *B*. If $\omega\in A_{p}$, then there exists $\delta>0$ such that

2.1 $$ \frac{\omega(E)}{\omega(B)}\lesssim \biggl(\frac{|E|}{|B|} \biggr)^{\delta} $$

for any measurable subset *E* of a ball *B*. Since the $A_{p}$ classes are increasing with respect to *p*, we use the following notation $A_{\infty}=\bigcup_{p>1}A_{p}$. $A\lesssim B$ means $A\leq CB$, where *C* is a positive constant independent of the main parameters. For $\lambda>0$ and a ball $B\subset\mathbb{R}^{n}$, we write *λB* for the ball with same center as *B* and radius *λ* times radius of *B*.

Now we give the definition of $A_{\vec{p}}$ condition.

### Definition 2.1

[2]

Let $1\leqslant p_{j}<\infty$ for $j=1,\ldots,m$, $\vec{p}=(p_{1},\ldots ,p_{m})$ and $\frac{1}{p}=\frac{1}{p_{1}}+\cdots+\frac{1}{p_{m}}$. Given $\vec{\omega}=(\omega_{1},\ldots,\omega_{m})$, set

$$ v_{\vec{\omega}}=\prod_{j=1}^{m} \omega_{j}^{p/p_{j}}. $$

We say that $\vec{\omega}\in A_{\vec{p}}$ if

$$ \sup_{B} \biggl(\frac{1}{|B|} \int_{B}v_{\vec{\omega}} \biggr)^{1/p}\prod _{j=1}^{m} \biggl(\frac{1}{|B|} \int_{B}\omega _{j}^{1-p'_{j}} \biggr)^{1/p'_{j}}< \infty. $$

$p'$ is the conjugate index of *p*. When $p_{j}=1$, denote $p'_{j}=\infty$, $(\frac{1}{|B|}\int_{B}\omega _{j}^{1-p{'}_{j}})^{1/p{'}_{j}}$ is understood as $(\inf_{B}\omega_{j})^{-1}$

Obviously, if $m=1$, $A_{\vec{p}}$ is the classical $A_{p}$ class. $A_{\vec{p}}$ has the following characterization.

### Lemma 2.2

[2]

*Let* $\vec{\omega}=(\omega_{1},\ldots,\omega_{m})$. *Then* $\vec {\omega}\in A_{\vec{p}}$ *if and only if*

$$ \omega_{j}^{1-p{'}_{j}}\in A_{mp{'}_{j}} \quad \textit{and} \quad v_{\vec {\omega}}\in A_{mp}, $$

*where the condition* $\omega_{j}^{1-p{'}_{j}}\in A_{mp_{j}{'}}$ *is understood as* $\omega_{j}^{1/m}\in A_{1}$ *in the case* $p_{j}=1$.

### Lemma 2.3

[2]

*Assume that* $\vec{\omega}=(\omega_{1},\ldots,\omega_{m})$ *satisfies* $A_{\vec{p}}$ *condition*. *Then there exists a finite constant* $r>1$ *such that* $\vec{\omega}\in A_{\vec{p}/r}$.

In order to prove the results for commutators, we need the following properties of *BMO*. For $b\in \mathit{BMO}$, $1< p<\infty$ and $\omega\in A_{\infty}$, we get

2.2 $$ \|b\|_{\mathit{BMO}}\sim\sup_{B} \biggl(\frac{1}{|B|} \int _{B}\bigl\vert b(x)-b_{B}\bigr\vert ^{p}\, \mathrm{d}x \biggr)^{1/p} $$

and for all balls *B*,

2.3 $$ \biggl(\frac{1}{\omega(B)} \int_{B}\bigl\vert b(x)-b_{B}\bigr\vert ^{p} \omega(x) \, \mathrm{d}x \biggr)^{1/p}\leq C\|b \|_{\mathit{BMO}}. $$

For all nonnegative integers *k*, we obtain

2.4 $$ |b_{2^{k+1}B}-b_{B}|\leq C(k+1)\|b \|_{\mathit{BMO}}, $$

where $\omega(B)=\int_{B}\omega(x)\, \mathrm{d}x$, $b_{B}=\frac {1}{|B|}\int_{B}b(x)\, \mathrm{d}x$ (see [4]).

## Proof of the main results

### Proof of Theorem 1.1

(1) Let $B=B(y,r)$ be a ball of $\mathbb{R}^{n}$, $f_{i}=f_{i}\chi _{2B}+f_{i}\chi_{(2B)^{c}}$ and denote $f_{i}\chi_{2B}$ by $f_{i}^{0}$ and $f_{i}\chi_{(2B)^{c}}$ by $f_{i}^{\infty}$ ($i=1,\ldots,m$), $\chi_{E}$ denotes the characteristic function of set *E*. For $x\in B(y,r)$, we have

$$\begin{aligned} \bigl\vert T\vec{f}(x)\bigr\vert \leqslant&\bigl\vert T \bigl(f_{1}^{0},\ldots,f_{m}^{0}\bigr) (x)\bigr\vert +\sum_{\alpha_{1},\ldots ,\alpha_{m}\in\{0,\infty\}}\bigl\vert T \bigl(f{_{1}^{\alpha_{1}}},\ldots,f{_{m}^{\alpha_{m}}} \bigr) (x)\bigr\vert \\ &{}+\bigl\vert T\bigl(f{_{1}^{\infty}}, \ldots,f{_{m}^{\infty}}\bigr) (x)\bigr\vert \\ = &I+\mathit{II}+\mathit{III}, \end{aligned}$$

where $\alpha_{1},\ldots,\alpha_{m}$ are not all equal to 0 or ∞ at the same time. We first estimate *III*. Since $2^{k-1}r\leqslant|x-y_{i}|\leqslant2^{k+2}r$, we have

3.1 $$\begin{aligned} \mathit{III} =& \biggl\vert \int_{(\mathbb{R}^{n}\diagdown2B )^{m}}\frac {f_{1}(y_{1})\cdots f_{m}(y_{m})}{ (\sum_{i=1}^{m}|x-y_{i}| )^{mn}}\,\mathrm{d}\vec{y}\biggr\vert \\ \leqslant& \int_{(\mathbb{R}^{n}\diagdown2B )^{m}}\frac {|f_{1}(y_{1})\cdots f_{m}(y_{m})|}{ (\sum_{i=1}^{m}|x-y_{i}| )^{mn}}\,\mathrm{d}\vec{y} \\ =& \sum_{k=1}^{\infty} \int_{{(2^{k+1}B\diagdown2^{k}B)^{m}}}\frac{|f_{1}(y_{1})\cdots f_{m}(y_{m})|}{ (\sum_{i=1}^{m}|x-y_{i}| )^{mn}}\,\mathrm{d}\vec{y} \\ \lesssim& \sum_{k=1}^{\infty}\prod _{i=1}^{m} \int _{{2^{k+1}{B}}\diagdown2^{k}{B}}\frac{|f_{i}(y_{i})|}{ |x-y_{i}|^{n}}\,\mathrm{d}y_{i} \\ \lesssim& \sum_{k=1}^{\infty} \frac{1}{|2^{k+1}B|^{m}}\prod_{i=1}^{m} \int_{2^{k+1}{B}}\bigl\vert f_{i}(y_{i})\bigr\vert \,\mathrm{d} {y_{i}} , \end{aligned}$$

the Hölder inequality gives us that

3.2 $$ \int_{2^{k+1}B}\bigl\vert f_{i}(y_{i})\bigr\vert \,\mathrm{d} {y_{i}}\leqslant \biggl( \int_{2^{k+1}B}\bigl\vert f(y_{i})\bigr\vert ^{p_{i}}\omega_{i}(y_{i}) \,\mathrm {d}y_{i} \biggr)^{1/p_{i}} \biggl( \int_{2^{k+1}B}\omega (y_{i})^{-p{'}_{i}/p_{i}} \, \mathrm{d}y_{i} \biggr)^{1/p{'}_{i}}. $$

By the definition of $A_{\vec{p}}$ condition, we obtain

3.3 $$ \mathit{III}\lesssim \sum_{k=1}^{\infty} \frac {1}{(\int_{2^{k+1}B}v_{\vec{\omega}})^{1/p}}\prod_{i=1}^{m}\| f_{i}\chi_{2^{k+1}B}\|_{L^{p_{i}}(\omega_{i})} . $$

For *II*, we just consider this case: $\alpha_{i}=\infty$ for $i=1,\ldots,l$ and $\alpha_{j}=0$ for $j=l+1,\ldots,m$,

3.4 $$\begin{aligned}& \bigl\vert T\bigl(f_{1}^{\infty},\ldots,f_{l}^{\infty},f_{l+1}^{0}, \ldots ,f_{m}^{0}\bigr) (x)\bigr\vert \\& \quad = \biggl\vert \int_{(\mathbb{R}^{n}\diagdown2B)^{l}} \int_{(2B)^{m-l}} \frac{f_{1}(y_{1})\cdots f_{m}(y_{m})}{(\sum^{m}_{i=1}|x-y_{i}|)^{mn}}\,\mathrm{d}\vec{y}\biggr\vert \\& \quad \leqslant \int_{(\mathbb{R}^{n}\diagdown2B)^{l}} \int _{(2B)^{m-l}} \frac{|f_{1}(y_{1})\cdots f_{m}(y_{m})|}{(\sum^{m}_{i=1}|x-y_{i}|)^{mn}}\,\mathrm{d}\vec{y} \\& \quad \lesssim \prod_{i=l+1}^{m} \int_{2B}\bigl|f_{i}(y_{i})\bigr| \,\mathrm {d}y_{i}\sum^{\infty}_{k=1} \frac{1}{|2^{k+1}B|^{m}} \prod_{i=1}^{l} \int_{2^{k+1}B\diagdown2^{k}B}\bigl|f_{i}(y_{i})\bigr| \,\mathrm{d} {y_{i}} \\& \quad \lesssim \sum^{\infty}_{k=1} \frac{1}{|2^{k+1}B|^{m}} \prod_{i=1}^{m} \int_{2^{k+1}B}\bigl\vert f_{i}(y_{i})\bigr\vert \,\mathrm {d} {y_{i}} . \end{aligned}$$

By (3.2) and the definition of $A_{\vec{p}}$ condition, we have

3.5 $$ \mathit{II}\lesssim\sum_{k=1}^{\infty} \frac{1}{(\int _{2^{k+1}B}v_{\vec{\omega}})^{1/p}}\prod_{i=1}^{m} \|f_{i}\chi _{2^{k+1}B}\|_{L^{p_{i}}(\omega_{i})} . $$

Combining all the cases together, we obtain

3.6 $$\begin{aligned} \bigl\vert T\vec{f}(x)\bigr\vert \lesssim&\biggl\vert \int_{(2B)^{m}}K(x,y_{1},\ldots ,y_{m})f_{1}(y_{1}) \cdots f_{m}(y_{m})\,\mathrm{d}\vec{y}\biggr\vert \\ &{}+\sum_{k=1}^{\infty}\frac{1}{(\int_{2^{k+1}B}v_{\vec{\omega }})^{1/p}} \prod_{i=1}^{m}\|f\chi_{2^{k+1}B} \|_{L^{p_{i}}(\omega_{i})}. \end{aligned}$$

Taking $L^{p}(v_{\vec{\omega}})$ norm on the ball $B(y,r)$ on both sides of (3.6), by (1.2), we get

3.7 $$\begin{aligned} \|T\vec{f}\chi_{B(y,r)}\|_{L^{p}(v_{\vec{\omega}})} \lesssim&\prod _{i=1}^{m}\|f_{i} \chi_{B(y,2r)}\| _{L^{p_{i}}(\omega_{i} )} \\ &{}+\sum_{k=1}^{\infty}\frac{(\int_{B}v_{\vec{\omega }})^{1/p}}{(\int_{2^{k+1}B}v_{\vec{\omega}})^{1/p}} \prod_{i=1}^{m}\| f_{i} \chi_{2^{k+1}B}\|_{L^{p_{i}}(\omega_{i})}. \end{aligned}$$

Multiplying both sides of (3.7) by $v_{\vec{\omega }}(B)^{1/\alpha-1/q-1/p}$, by Lemma 2.2 and (2.1), we obtain

3.8 $$\begin{aligned}& v_{\vec{\omega}}(B)^{1/\alpha-1/q-1/p}\|T\vec{f}\chi_{B(y,r)}\| _{L^{p}(v_{\vec{\omega}})} \\& \quad \lesssim \sum_{k=0}^{\infty} \frac{1}{2^{nk\delta(1/\alpha-1/q)}} v_{\vec{\omega}}\bigl(2^{k+1}B\bigr)^{1/\alpha-1/q-1/p} \prod_{i=1}^{m}\|f_{i} \chi_{B(y,2^{k+1}r)}\|_{L^{p_{i}}(\omega_{i})} . \end{aligned}$$

For $\frac{1}{p_{1}/p}+\frac{1}{p_{2}/p}+\cdots+\frac {1}{p_{m}/p}=1$, we have

$$\begin{aligned}& \bigl\Vert v_{\vec{\omega}}(B)^{1/\alpha-1/q-1/p}\Vert T\vec{f} \chi_{B(y,r)}\Vert _{L^{p}(v_{\vec{\omega}})}\bigr\Vert _{L^{q}(R^{n})} \\& \quad \lesssim \sum_{k=0}^{\infty} \frac{1}{2^{nk\delta(1/\alpha -1/q)}}\prod_{i=1}^{m}\bigl\Vert \omega_{i}\bigl(2^{k+1}B\bigr)^{p/\alpha p_{i}-1/p_{i}-p/qp_{i}} \Vert f_{i}\chi_{B(y,2^{k+1}r)}\Vert _{L^{p_{i}}(\omega_{i})}\bigr\Vert _{L^{qp_{i}/p}(R^{n})} \end{aligned}$$

by the Hölder inequality. Since $\sum_{k=0}^{\infty}\frac {1}{2^{nk\delta(1/\alpha-1/q)}}<\infty$, we obtain the expected result

$$ \|T\vec{f}\|_{(L^{p}(v_{\vec{\omega}}),L^{q})^{\alpha}}\lesssim \prod_{i=1}^{m} \|f_{i}\|_{({L^{p_{i}}(\omega_{i}),L^{qp_{i}/p}})^{\alpha p_{i}/p}}. $$

(2) For $\lambda>0$, by (3.6) and (1.3), we get

$$\begin{aligned}& \lambda v_{\vec{\omega}}\bigl(x\in B(y,r):\bigl\vert T\vec{f}(x)\bigr\vert > \lambda \bigr)^{1/p} \\& \quad \lesssim\prod_{i=1}^{m} \|f_{i}\chi_{B(y,2r)}\| _{L^{p_{i}}(\omega_{i})} +\sum_{k=1}^{\infty}\frac{(\int_{B}v_{\vec{\omega }})^{1/p}}{(\int_{2^{k+1}B}v_{\vec{\omega}})^{1/p}} \prod_{i=1}^{m}\| f_{i} \chi_{2^{k+1}B}\|_{L^{p_{i}}(\omega_{i})}. \end{aligned}$$

That is,

3.9 $$\begin{aligned}& \|Tf\chi_{B(y,r)}\|_{L^{p,\infty}(v_{\vec{\omega}})} \\& \quad \lesssim\prod _{i=1}^{m}\|f_{i}\chi_{B(y,2r)}\| _{L^{p_{i}}(\omega_{i} )} +\sum_{k=1}^{\infty}\frac{(\int_{B}v_{\vec{\omega }})^{1/p}}{(\int_{2^{k+1}B}v_{\vec{\omega}})^{1/p}} \prod_{i=1}^{m}\| f_{i} \chi_{2^{k+1}B}\|_{L^{p_{i}}(\omega_{i})}. \end{aligned}$$

Multiplying both sides of (3.9) by $v_{\vec{\omega }}(B)^{1/\alpha-1/q-1/p}$, we conclude as in the case (1). □

### Proof of Theorem 1.2

It suffices to prove $T_{\vec{b}}^{j}$. For $B=B(y,r)$, $x\in B$

$$\begin{aligned} T_{\vec{b}}^{j}(\vec{f}) (x) =&T_{\vec{b}}^{j}( \vec{f}\chi _{2B}) (x) +\sum_{\alpha_{1},\ldots,\alpha_{m}\in\{0,\infty\}} \bigl(b_{j}(x)T \bigl(f{_{1}^{\alpha_{1}}},\ldots,f_{j}^{ \alpha_{j}}, \ldots,f{_{m}^{\alpha_{m}}}\bigr) \\ &{}-T\bigl(f{_{1}^{\alpha_{1}}},\ldots,b_{j}f_{j}^{\alpha_{j}}, \ldots,f{_{m}^{\alpha _{m}}}\bigr) (x) \bigr) \\ &{}+b_{j}(x)T\bigl(f_{1}^{\infty}, \ldots,f_{j}^{\infty},\ldots,f_{m}^{\infty } \bigr)-T\bigl(f_{1}^{\infty},\ldots,b_{j}f_{j}^{\infty}, \ldots,f_{m}^{\infty }\bigr) (x) \\ = &I'+\mathit{II}'+\mathit{III}' , \end{aligned}$$

where $\alpha_{1},\ldots,\alpha_{m}$ are not all equal to 0 or ∞ at the same time. We first deal with $\mathit{III}'$.

3.10 $$\begin{aligned} \bigl\vert \mathit{III}'\bigr\vert \leq& \bigl\vert \bigl(b_{j}(x)-b_{B}\bigr)T\bigl(f_{1}^{\infty}, \ldots ,f_{j}^{\infty},\ldots,f_{m}^{\infty} \bigr)\bigr\vert \\ &{}+\bigl\vert T\bigl(f_{1}^{\infty},\ldots,(b_{j}-b_{B})f_{j}^{\infty}, \ldots ,f_{m}^{\infty}\bigr) (x)\bigr\vert \\ \leq& \bigl\vert \bigl(b_{j}(x)-b_{B}\bigr)\bigr\vert \sum_{k=1}^{\infty}\frac{1}{(\int _{2^{k+1}B}v_{\vec{\omega}})^{1/p}}\prod _{i=1}^{m}\|f_{i}\chi _{2^{k+1}B}\|_{L^{p_{i}}(\omega_{i})} \\ &{}+\sum_{k=1}^{\infty}\frac{|b_{2^{k+1}B}-b_{B}|}{(\int _{2^{k+1}B}v_{\vec{\omega}})^{1/p}} \prod_{i=1}^{m}\|f_{i}\chi _{2^{k+1}B}\|_{L^{p_{i}}(\omega_{i})} \\ &{}+\sum_{k=1}^{\infty}\frac{1}{|2^{k+1}B|^{m}} \int _{({2^{k+1}{B}})^{m}}{\prod_{i=1,i\neq j}^{m} \bigl\vert f_{i}(y_{i})f_{j}(y_{j}) \bigl(b_{j}(y_{j})-b_{2^{k+1}B} \bigr)\bigr\vert }\, \mathrm{d}\vec{y} . \end{aligned}$$

Select suitable $s>1$ to raise $\int_{({2^{k+1}{B}})^{m}}{\prod_{i=1,i\neq j}^{m}|f_{i}(y_{i})f_{j}(y_{j}) (b_{j}(y_{j})-b_{2^{k+1}B} )|}\,\mathrm{d}\vec{y}$ by the Hölder inequality and such that $\vec {\omega}\in A_{\vec{p}/s}$ by Lemma 2.3. Then characterization $A_{\vec{p}/s}$ and (2.3) yield

3.11 $$\begin{aligned}& \sum_{k=1}^{\infty}\frac{1}{\vert 2^{k+1}B\vert ^{m}} \int _{({2^{k+1}{B}})^{m}}{\prod_{i=1,i\neq j}^{m} \bigl\vert f_{i}(y_{i})f_{j}(y_{j}) \bigl(b_{j}(y_{j})-b_{2^{k+1}B} \bigr)\bigr\vert }\, \mathrm{d}\vec{y} \\& \quad \leq \sum^{\infty}_{k=1} \frac{1}{\vert 2^{k+1}B\vert ^{m/s}} \Biggl(\prod_{i=1,i\neq j}^{m} \int _{2^{k+1}B}\bigl\vert f_{i}(y_{i}) \bigr\vert ^{s} \,\mathrm{d} {y_{i}} \Biggr)^{1/s} \\& \qquad {}\times \biggl( \int_{2^{k+1}B}\bigl\vert \bigl(b_{j}(y_{j})-b_{2^{k+1}B} \bigr)f_{j}(y_{j})\bigr\vert ^{s} \, \mathrm{d}y_{j} \biggr)^{1/s} \\& \quad \leq \sum^{\infty}_{k=1} \frac{1}{\vert 2^{k+1}B\vert ^{m/s}} \prod_{i=1,i\neq j}^{m} \biggl( \int _{2^{k+1}B}\bigl\vert f_{i}(y_{i}) \bigr\vert ^{p_{i}}\omega_{i}(y_{i}) \,\mathrm {d} {y_{i}} \biggr)^{1/p_{i}} \\& \qquad {}\times \biggl( \int_{2^{k+1}B}\omega_{i}(y_{i})^{-s/(p_{i}-s)} \,\mathrm{d} {y_{i}} \biggr)^{(p_{i}-s)/p_{i}s} \\& \qquad {} \times \biggl( \int _{2^{k+1}B}\bigl\vert b_{j}(y_{j})-b_{2^{k+1}B} \bigr\vert ^{p_{j}s/(p_{j}-s)}\omega _{i}(y_{j})^{-s/(p_{j}-s)} \,\mathrm{d}y_{j} \biggr)^{1/s} \\& \qquad {}\times \biggl( \int_{2^{k+1}B}\bigl\vert f_{j}(y_{j})\bigr\vert ^{p_{j}}\omega_{j}(y_{j}) \,\mathrm {d} {y_{j}} \biggr)^{1/p_{j}} \\& \quad \lesssim \|b_{j}\|_{\mathit{BMO}}\sum _{k=1}^{\infty}\frac{1}{(\int _{2^{k+1}B}v_{\vec{\omega}})^{1/p}}\prod _{i=1}^{m}\|f_{i}\chi _{2^{k+1}B} \|_{L^{p_{i}}(\omega_{i})} . \end{aligned}$$

So we have

3.12 $$\begin{aligned} \bigl\vert \mathit{III}'\bigr\vert \leq& \bigl\vert \bigl(b_{j}(x)-b_{B}\bigr)\bigr\vert \sum _{k=1}^{\infty}\frac {1}{(\int_{2^{k+1}B}v_{\vec{\omega}})^{1/p}}\prod _{i=1}^{m}\| f_{i}\chi_{2^{k+1}B} \|_{L^{p_{i}}(\omega_{i})} \\ &{}+\sum_{k=1}^{\infty}\frac{|b_{2^{k+1}B}-b_{B}|}{(\int _{2^{k+1}B}v_{\vec{\omega}})^{1/p}} \prod_{i=1}^{m}\|f_{i}\chi _{2^{k+1}B}\|_{L^{p_{i}}(\omega_{i})} \\ &{}+\|b_{j}\|_{\mathit{BMO}}\sum_{k=1}^{\infty} \frac{1}{(\int_{2^{k+1}B}v_{\vec {\omega}})^{1/p}}\prod_{i=1}^{m} \|f_{i}\chi_{2^{k+1}B}\| _{L^{p_{i}}(\omega_{i})} . \end{aligned}$$

For $\mathit{II}'$, we just consider this case: $\alpha_{i}=\infty$ for $i=1,\ldots,l$ and $\alpha_{j}=0$ for $j=l+1,\ldots,m$. There are two cases:

$$\begin{aligned} \begin{aligned} &b_{j}(x)T\bigl(f_{1}^{\infty},\ldots,f_{j}^{\infty}, \ldots,f_{l}^{\infty },f_{l+1}^{0}, \ldots,f_{m}^{0}\bigr) \\ &\quad {}- T\bigl(f_{1}^{\infty},\ldots,b_{j}f_{j}^{\infty}, \ldots,f_{l}^{\infty },f_{l+1}^{0}, \ldots,f_{m}^{0}\bigr) (x) \end{aligned} \end{aligned}$$

or

$$\begin{aligned}& b_{j}(x)T\bigl(f_{1}^{\infty},\ldots,f_{l}^{\infty},f_{l+1}^{0}, \ldots ,f_{j}^{0},\ldots,f_{m}\bigr) \\& \quad {}- T\bigl(f_{1}^{\infty},\ldots,f_{l}^{\infty},f_{l+1}^{0}, \ldots ,b_{j}f_{j}^{0},\ldots,f_{m}^{0} \bigr) (x). \end{aligned}$$

We just consider the following case:

3.13 $$\begin{aligned}& \bigl\vert b_{j}(x)T\bigl(f_{1}^{\infty}, \ldots,f_{j}^{\infty},\ldots,f_{l}^{\infty },f_{l+1}^{0}, \ldots,f_{m}^{0}\bigr) \\& \qquad {}-T\bigl(f_{1}^{\infty},\ldots,b_{j}f_{j}^{\infty}, \ldots,f_{l}^{\infty },f_{l+1}^{0}, \ldots,f_{m}^{0}\bigr) (x)\bigr\vert \\& \quad \leq \bigl\vert \bigl(b_{j}(x)-b_{B}\bigr)T \bigl(f_{1}^{\infty},\ldots,f_{j}^{\infty}, \ldots ,f_{l}^{\infty},f_{l+1}^{0}, \ldots,f_{m}^{0}\bigr)\bigr\vert \\& \qquad {} +\bigl\vert T\bigl(f_{1}^{\infty}, \ldots,(b_{j}-b_{B})f_{j}^{\infty},\ldots ,f_{l}^{\infty},f_{l+1}^{0}, \ldots,f_{m}^{0}\bigr) (x)\bigr\vert \\& \quad \leq \bigl\vert \bigl(b_{j}(x)-b_{B}\bigr)\bigr\vert \sum_{k=1}^{\infty}\frac{1}{(\int _{2^{k+1}B}v_{\vec{\omega}})^{1/p}} \prod_{i=1}^{m}\|f_{i}\chi _{2^{k+1}B}\|_{L^{p_{i}}(\omega_{i})} \\& \qquad {} +\sum_{k=1}^{\infty} \frac{|b_{2^{k+1}B}-b_{B}|}{(\int _{2^{k+1}B}v_{\vec{\omega}})^{1/p}}\prod_{i=1}^{m} \|f_{i}\chi _{2^{k+1}B}\|_{L^{p_{i}}(\omega_{i})} \\& \qquad {} +\sum_{k=1}^{\infty} \frac{\prod_{i=l+1}^{m}\int _{2B}|f_{i}(y_{i})| \,\mathrm{d}y_{i} }{|2^{k+1}B|^{m}} \int_{({2^{k+1}{B}})^{m}}{\prod_{i=1,i\neq j}^{l} \bigl\vert f_{i}(y_{i})f_{j}(y_{j}) \bigl(b_{j}(y_{j})-b_{2^{k+1}B} \bigr)\bigr\vert }\, \mathrm {d}\vec{y} . \end{aligned}$$

The estimate for

$$\sum_{k=1}^{\infty}\frac{\prod_{i=l+1}^{m}\int _{2B}|f_{i}(y_{i})| \,\mathrm{d}y_{i} }{|2^{k+1}B|^{m}} \int_{({2^{k+1}{B}})^{m}}{\prod_{i=1,i\neq j}^{l} \bigl\vert f_{i}(y_{i})f_{j}(y_{j}) \bigl(b_{j}(y_{j})-b_{2^{k+1}B} \bigr)\bigr\vert } \,\mathrm {d}\vec{y} $$

is similar to (3.11). We get

3.14 $$\begin{aligned}& \sum_{k=1}^{\infty}\frac{\prod_{i=l+1}^{m}\int _{2B}|f_{i}(y_{i})| \,\mathrm{d}y_{i} }{|2^{k+1}B|^{m}} \int_{({2^{k+1}{B}})^{m}}{\prod_{i=1,i\neq j}^{l} \bigl\vert f_{i}(y_{i})f_{j}(y_{j}) \bigl(b_{j}(y_{j})-b_{2^{k+1}B} \bigr)\bigr\vert }\, \mathrm {d}\vec{y} \\& \quad \leq \sum^{\infty}_{k=1} \frac{1}{|2^{k+1}B|^{m}} \prod_{i=1,i\neq j}^{m} \int_{2^{k+1}B}\bigl\vert f_{i}(y_{i})\bigr\vert \,\mathrm {d} {y_{i}}\times \int_{2^{k+1}B}\bigl\vert b_{j}(y_{j})-b_{2^{k+1}B} \bigr\vert f(y_{j}) \,\mathrm {d}y_{j} \\& \quad \lesssim \|b_{j}\|_{\mathit{BMO}}\sum _{k=1}^{\infty}\frac{1}{(\int _{2^{k+1}B}v_{\vec{\omega}})^{1/p}}\prod _{i=1}^{m}\|f\chi _{2^{k+1}B}\|_{L^{p_{i}}(\omega_{i})} , \end{aligned}$$

so we have

3.15 $$\begin{aligned} \bigl\vert T_{\vec{b}}^{j}(\vec{f}) (x)\bigr\vert \leq&\bigl\vert T_{\vec{b}}^{j}(\vec{f}\chi _{2B}) (x)\bigr\vert + \bigl\vert \bigl(b_{j}(x)-b_{B}\bigr)\bigr\vert \sum_{k=1}^{\infty}\frac{1}{(\int _{2^{k+1}B}v_{\vec{\omega}})^{1/p}}\prod _{i=1}^{m}\|f\chi _{2^{k+1}B} \|_{L^{p^{i}}(\omega_{i})} \\ &{}+\sum_{k=1}^{\infty}\frac{|b_{2^{k+1}B}-b_{B}|}{(\int _{2^{k+1}B}v_{\vec{\omega}})^{1/p}} \prod_{i=1}^{m}\|f_{i}\chi _{2^{k+1}B}\|_{L^{p^{i}}(\omega_{i})} \\ &{}+\|b_{j}\|_{\mathit{BMO}}\sum_{k=1}^{\infty} \frac{1}{(\int_{2^{k+1}B}v_{\vec {\omega}})^{1/p}}\prod_{i=1}^{m} \|f_{i}\chi_{2^{k+1}B}\| _{L^{p^{i}}(\omega_{i})} . \end{aligned}$$

Take $L^{p}(v_{\vec{\omega}})$ norm on the ball $B(y,r)$ on both sides of (3.15). By (1.5), (2.3), (2.4), we have

3.16 $$\begin{aligned}& \bigl\Vert T_{\vec{b}}^{j}(\vec{f}) \chi_{B(y,r)}\bigr\Vert _{L^{p}(v_{\vec{\omega }})} \\& \quad \lesssim \|b_{j}\|_{\mathit{BMO}}\prod _{i=1}^{m}\|f_{i}\chi_{B(y,2r)}\| _{L^{p_{i}}(\omega_{i} )} \\& \qquad {}+\|b_{j}\|_{\mathit{BMO}}\sum_{k=1}^{\infty} \frac{k(\int_{B}v_{\vec{\omega }})^{1/p}}{(\int_{2^{k+1}B}v_{\vec{\omega}})^{1/p}}\prod_{i=1}^{m}\| f_{i}\chi_{2^{k+1}B}\|_{L^{p_{i}}(\omega_{i})}. \end{aligned}$$

Multiplying both sides of (3.16) by $v_{\vec{\omega }}(B)^{1/\alpha-1/q-1/p}$, by Lemma 2.2 and (2.1), we obtain

3.17 $$\begin{aligned}& v_{\vec{\omega}}(B)^{1/\alpha-1/q-1/p}\bigl\Vert T_{\vec{b}}^{j}(\vec {f})\chi_{B(y,r)}\bigr\Vert _{L^{p}(v_{\vec{\omega}})} \\& \quad \lesssim \sum_{k=0}^{\infty} \frac{(k+1)\|b_{j}\|_{\mathit{BMO}}}{2^{nk\delta (1/\alpha-1/q)}} v_{\vec{\omega}}\bigl(2^{k+1}B\bigr)^{1/\alpha-1/q-1/p} \prod_{i=1}^{m}\|f_{i} \chi_{B(y,2^{k+1}r)}\|_{L^{p_{i}}(\omega_{i})}. \end{aligned}$$

Next the proof is similar to Theorem 1.1 of (1), we get

$$ \bigl\Vert T_{\vec{b}}^{j}(\vec{f})\bigr\Vert _{(L^{p}(v_{\vec{\omega }}),L^{q})^{\alpha}}\lesssim\|\vec{b}\|_{(\mathit{BMO})^{m}}\prod _{i=1}^{m}\| f_{i}\|_{({L^{p_{i}}(\omega_{i}),L^{qp_{i}/p}})^{\alpha p_{i}/p}}. $$

 □
